# GWAS meta-analysis provides new insights into uveal melanoma risk

**DOI:** 10.1038/s41416-026-03499-7

**Published:** 2026-06-16

**Authors:** Matthew D’Mellow, Huanwei Wang, Jane M. Palmer, Kari Hemminki, Colleen M. Cebulla, Aaron B. Beasley, Nikolaos E. Bechrakis, Antonia L. Pritchard, Karin W. Wadt, Peter A. Johansson, Lenha Mobuchon, Samantha Barlow, Kelly Brooks, Timothy Beckman, Catherine M. Olsen, Sunil K. Warrier, Lindsey Byrne, Helen Kalirai, Colette Mustard, Nathan Ingold, Asta Försti, Timothy Isaacs, G. J. M. Shanika R. Jayasinghe, William J. Glasson, Gayle Williamson, Lindsay A. McGrath, Ngoc-Quynh Le, Vikas Chadha, Elin S. Gray, Kevin M. Brown, Paul Cauchi, Hauke Thomsen, Julie Connolly, Stuart MacGregor, David C. Whiteman, Jens F. Kiilgaard, Marc-Henri Stern, Sarah E. Coupland, Mohamed H. Abdel-Rahman, Michael Zeschnigk, Nick Hayward, Matthew H. Law

**Affiliations:** 1 Cancer Research Program, QIMR Berghofer Medical Research Institute, Brisbane, QLD Australia; 2 Terrace Eye Centre, Brisbane, QLD Australia; 3 Population Health Research Program, QIMR Berghofer Medical Research Institute, Brisbane, QLD Australia; 4 Faculty of Health, Queensland University of Technology, Brisbane, QLD Australia; 5 Charles University Medical School, Pilsen, Czechia; 6 German Cancer Research Center (DKFZ), Heidelberg, Germany; 7 Department of Ophthalmology and Visual Sciences, Havener Eye Institute, The Ohio State University Wexner Medical Center, Columbus, OH USA; 8 Centre for Precision Health, Edith Cowan University, Joondalup, WA Australia; 9 Institute of Human Genetics, Universitätsklinikum Essen, Essen, Germany; 10 Biomedical Sciences, University of the Highlands and Islands, Inverness, UK; 11 Rigshospitalet, Copenhagen, Denmark; 12 Faculty of Health, Medicine and Behavioural Sciences, University of Queensland, Brisbane, QLD Australia; 13 PSL University, Institut Curie, Paris, France; 14 Department of Eye and Vision Science, University of Liverpool, Liverpool, UK; 15 Division of Human Genetics, Department of Internal Medicine, The Ohio State University Wexner Medical Center, Columbus, OH USA; 16 Hopp Children’s Cancer Center (KiTZ), Heidelberg, Germany; 17 Perth Retina, Perth, WA Australia; 18 University of Western Australia, Crawley, WA Australia; 19 Royal Perth Hospital, Perth, WA Australia; 20 Tennent Institute of Ophthalmology, Gartnavel General Hospital, Glasgow, UK; 21 Harry Perkins Institute of Medical Research, Nedlands, WA Australia; 22 National Cancer Institute, National Institutes of Health, Bethesda, ML USA; 23 MSB Medical School Berlin, Berlin, Germany; 24 Department of Clinical Medicine, Copenhagen University, Copenhagen, Denmark

**Keywords:** Eye cancer, Cancer genomics, Genome-wide association studies, Oncogenes, Risk factors

## Abstract

**Objective:**

The aim of this research is to identify germline genetic variants that predispose to uveal melanoma (UM) using data from nine studies involving 5839 individuals with UM (3853 novel) and 349,863 healthy controls.

**Methods:**

Five novel UM genome-wide association studies (GWAS) were performed and included for meta-analysis with four previously published UM GWAS. A fixed-effects inverse-variance weighted (IVW) meta-analysis was performed by combining data from these nine UM case-control cohorts. A follow-up transcriptome-wide association study (TWAS) was conducted to identify candidate target genes at UM risk loci. Genetic correlations with melanoma-related phenotypes were measured to elucidate UM’s genetic architecture.

**Results:**

We identify nine linkage disequilibrium (LD)-independent loci (three novel) with an IVW *P* value of less than 5 × 10^−8^. TWAS analysis indicates five potential target genes, including *MOB3B*, *RBAK*, and *MTSS1*, which have established links to multiple cancer types. We note a significant genetic correlation (rg = 0.31, *P* = 0.01) between UM and cutaneous melanoma (CM), and a non-significant but consistent correlation with naevus count (rg = 0.25, *P* = 0.08).

**Conclusions:**

This meta-analysis offers new insights into the genetic architecture of UM, highlights potential therapeutic targets, and explores the genetic relationship with CM and skin pigmentation.

## Background

Uveal melanoma (UM) is an eye cancer that presents most often in the choroid (~90% of all cases) and more rarely in the ciliary body (~6%) or iris (~4%) [1]. UM affects 10 people per million per year in Northern Europe, six per million per year in countries with a large proportion of Northern European ancestry (including Australia and the USA), and one case per million per year in Asia [2]. While Australia’s high rates of cutaneous melanoma (CM) are due to ultraviolet (UV) light exposure, the somatic genetic mutations in most UM lack a UV-associated mutation signature. While this suggests sun exposure is not a major risk factor for UM, recent research shows that UV exposure may be implicated in iris UM specifically [3–6]. Although rare, UM is still the most common primary intraocular malignancy in adults, and despite effective local tumour control through radiological, surgical, and laser therapies [7, 8], approximately half of all UM cases metastasize within 10 years of the initial diagnosis. Metastatic spread is most often to the liver [9], with an extremely poor prognosis and a one-year survival of only ~15% [10]. Advancements in CM treatments have not translated to meaningful improvement in UM therapies, and currently, clinical trial participation is the only treatment option for non-HLA-A*02:01 positive patients with unresectable metastatic UM [11–16].

Current knowledge of genetic risk factors for UM centres around rare, highly penetrant mutations in genes including *BAP1* [17–19], *CDKN2A* [20], *MBD4* [21–23], *BRCA2* [24, 25], and others (*PALB2*, *MLH1*, *MSH6*, *CHEK2*, *SMARCE1*, *ATM*, *BRCA1*, and *CTNNA1*) identified by whole exome sequencing (WES) of UM patients with high risk of hereditary cancer [26]. While less is understood about the role of common, low-penetrance germline variants in UM, several loci (*OCA2*, *IRF4*, *CLPTM1L*/*TERT*, *TDP1, RP11-536I6.1, RREB1*, *XPO4*, and *IP6K1*) have been identified through candidate gene studies [27] and genome-wide association studies (GWAS) [28–31].

As only a limited number of genetic risk loci have been found for UM, and identifying genes responsible for UM development may provide targets for future treatments, we sought to further characterise the germline genetic architecture of UM. To this end, we genotyped large novel sample sets from Australia, Germany, Denmark, the USA and the UK, and combined this with data from French, UK Biobank and FinnGen cohorts. Our meta-analysis doubles the case number of the most recent GWAS by Mies et al. in 2025 [31]. Following this, we used transcriptome-wide association study (TWAS) data from skin and melanocytes to identify target genes, and we explored the overlap between UM and potentially related traits to better understand the genetic architecture of the disease.

## Methods

### Study populations

A full description of all cohorts is provided in the supplementary methods. For the UKBB and FinnGen cohorts, cases were drawn from cancer registry, hospital or death record data based on ICD10 codes C69.3 (malignant neoplasm: choroid) and C69.4 (malignant neoplasm: ciliary body). For all other cohorts, clinical diagnoses were made by trained ocular oncologists and based on established international diagnostic standards [1]. For newly genotyped samples, germline DNA was extracted using standard methods from whole blood or saliva provided by UM patients from two Australian centres including Queensland Ocular Oncology Service, Terrace Eye Centre (*N* = 799) and the Edith Cowan University (*N* = 97); from Germany the Institut für Humangenetik, Universitätsklinikum Essen (*N* = 1536); from the USA the Ohio State University Comprehensive Cancer Center (*N* = 565), two UK centres including the Liverpool Ocular Oncology Research Group (*N* = 480) and the University of the Highlands and Island, Scotland (*N* = 34); and from Denmark, the Rigshospitalet Copenhagen (*N* = 346) (Table 1). All samples were collected with written informed consent following relevant local institutional review board approval. These DNA samples were genotyped on the Infinium Global Screening Array-24 (GSA) v2.0 BeadChip (Illumina, San Diego, USA).

**Table 1:** Cohort details for GWAS included in UM meta-analysis

Cohort name	Country (Region)	Case definition^a^	Cases pre-QC	Cases post-QC	Case source	Controls pre-QC	Controls post-QC	Control source	Published
AUSUK	Combined		1410	1338	See subset	24,943	18,780	QSKIN	N/A
	Australia (QLD)	Clinical diagnosis	799^b^	761^b^	Queensland Ocular Oncology Service, Terrace Eye Centre				
	Australia (WA)	Clinical diagnosis	97^b^	94^b^	Edith Cowan University				
	UK (England)	Clinical diagnosis	480^b^	451^b^	Liverpool Ocular Oncology Research Group				
	UK (Scotland)	Clinical diagnosis	34^b^	32^b^	University of the Highlands and Islands				
German	Germany	Clinical diagnosis	1536	1430	Institut für Humangenetik, Universitätsklinikum Essen	17,284	3927	CARTaGENE (17k batch 1)	N/A
USA	USA	Clinical diagnosis	565	534	Ohio State University Comprehensive Cancer Center	4178	1973	CARTaGENE (4224 batch)	N/A
Danish	Denmark	Clinical diagnosis	346	340	Rigshospitalet Copenhagen	17,284	2991	CARTaGENE (17k batch 2)	N/A
French (GSA)	France	Hospital^d^	946	889	Institut Curie	5237	2312	CARTaGENE (5300 batch)	PMID: 34424336
French (Omni5)	France	Hospital^d^	279	265	Institut Curie	7639	2792	PEGASUS	PMID: 28781888
English	UK (England)	Clinical diagnosis	648	590	Liverpool Ocular Oncology Research Group	5677	5199	UKWTCC	PMID: 31626034
UKBB	UK	Cancer registry^c^	225	211	UK Biobank	300,000	12,005^e^	UK Biobank	N/A
FinnGen	Finland	Hospital or Death record^c^	242	242	FinnGen Biobank	392,423	299,884	FinnGen Biobank	https://r9.finngen.fi/
Total:			6097	5839		774,665	349,863		

*UKWTCCC* UK Wellcome Trust Case Control Consortium.

^a^Records if the samples are from cancer registry data, hospital records, etc.

^b^Counts for each included set are provided for reference, with only the combined, final amount contributing to the total sample size.

^c^Record matching ICD10 C69.3 or C69.4.

^d^Consecutive series of newly diagnosed uveal melanoma cases at Institut Curie.

^e^To limit computation overhead and the limited gain in power with excessive control counts, only a subset of UKBB was used.

In addition to these newly genotyped samples, published individual-level genotype data were contributed by the Institut Curie, France (279 genotyped on the Illumina HumanOmni5 platform, 946 genotyped on the Infinium GSA 24 v1.0, see Table 1) [28–30].

Controls were drawn from individuals with North-West European ancestry from the CARTaGENE biobank (Canada), the QSkin study (Australia), and the Pegasus study (USA) (Table 1). Controls from QSkin and Pegasus were matched with cases using principal components analysis (PCA), which was also used to exclude ancestry outliers. As CARTaGENE samples include a diverse collection of ancestries, individuals with North-West European ancestry were first identified using a random forest model trained on UK Biobank (UKBB)-derived principal components and self-reported ethnic backgrounds [32]. After this selection step, PCA was applied to the CARTaGENE controls and UM cases as done for other sets. The CARTaGENE controls were divided into four separate and independent control sets to provide sufficient matches for several case cohorts while still maintaining adequate statistical power.

A GWAS for UM was also performed in the UKBB using Regenie (see supplementary methods) [33, 34]. Finally, published or publicly available summary statistics from GWAS involving populations drawn from FinnGen (release 9, endpoint CD2_UVEAMELANOMA_EXALLC) [35], the Liverpool Ocular Oncology Research Group [29], and the UK Wellcome Trust Case Control Consortium [36] were also used for meta-analysis (Table 1).

### Quality control for genotype data

Quality control (QC) measures for genotype data for individuals included a maximum genotype missingness rate of 0.03, nominated vs genotype-estimated sex mismatches and ambiguous sex calls, genotype heterozygosity >0.05 from the mean, and a maximum interrelatedness of 0.1875 calculated through identity by descent (IBD). Ancestry outliers from each cohort were identified via PCA and were excluded from the analysis.

Single-nucleotide polymorphism (SNP) level QC measures included a minimum call rate of 0.97, a minor allele frequency (MAF) threshold of 0.01, and a Hardy-Weinberg equilibrium (HWE) exact test *P* value threshold of 5e-10. Case and control sets were cleaned separately before merging and undergoing repeated QC. SNP missingness was also tested between sample groups to reduce the chance of any resultant SNP associations being caused by subtle differences between sets. SNP alignment and annotation were harmonised using the 1000 Genomes Project reference panel.

### Imputation and GWAS analysis

The cleaned genotype data for the Australian, English, Scottish, French, USA, Danish and German datasets (Table 1) were imputed through TOPMed Imputation Server Minimac v4.1.6 using TOPMed reference panel version r3. The uploaded unphased hg19 genotype was converted via liftover to hg38 and phased by Eagle (v2.4). SNP association *P* values and odds ratios (OR) were calculated with logistic-Firth hybrid regression analysis via PLINK 1.9 [37], using sex and the first 10 principal components as covariates to reduce confounding. A dummy variable was also used as a covariate in the Australian dataset to control for possible batch effects between genotyping centres.

Following standard QC recommended for the UKBB, imputed genotype data for 211 UM cases and 12,000 controls were analysed via logistic regression with Firth correction using Regenie (supplementary methods) [33]. Sex, age and principal components 1–10 were included as covariates.

Methods for estimating genomic inflation, heritability, genetic correlation and functional mapping are detailed in the supplementary methods.

### Meta-analysis

SNPs passing an MAF threshold of 0.01 and an imputation R-squared (*R*²) threshold of 0.3 from the nine GWAS datasets were used to perform a fixed- and random-effects inverse-variance weighted meta-analysis with PLINK v1.90b6.8 [38]. Conditional analysis was conducted to detect any secondary hits conditioning on the lead SNP in each locus using Genome-wide Complex Trait Analysis (GCTA, --cojo-slct option) with default options (*P* value threshold of 5e-08 and collinearity threshold of 0.9 [39].

### TWAS

The TWAS analysis was performed to identify the potential causal genes underlying the GWAS signals. Five eQTL datasets were used, including four from four different tissues from European samples from the 8th version of the GTEx cohort (i.e., Cells_Cultured_fibroblasts, Skin_Not_Sun_Exposed_Suprapubic, Skin_Sun_Exposed_Lower_leg, and Whole_Blood) [40], and one eQTL dataset from human melanocytes [41]. The TWAS software FUSION was used (FUSION.assoc_test.R), which integrated a COLOC analysis (--coloc_P option) [42]. With a Bonferroni method-corrected *P* value threshold of 1.2e-06 (0.05/41567, where 41567 is the total number of genes tested across five eQTL datasets) and a COLOC PP4 > 0.75 (the posterior probability of the hypothesis that only one shared SNP is associated with both gene expression and trait), we identified potential causal genes for five GWAS loci (Table 2 and Fig. s1). The locus plots were generated by FUSION software (FUSION.post_process.R).

### Ethical approval and consent to participate

This study was performed in accordance with the Declaration of Helsinki. All samples were collected with informed consent at each institute and shared as de-identified DNAs with QIMR Berghofer. Overall ethical approval was obtained from the ethics committee of QIMR Berghofer (P452). For details of specific cohorts’ approvals, see Supplementary Methods.

**Table 2:** TWAS Fusion results for uveal melanoma loci

Tissue	Gene	CHR	SNP	TWAS Z-score	TWAS P	COLOC PP4
GTExv8.EUR.Skin_Not_Sun_Exposed_Suprapubic	*CLPTM1L*	5	rs452384	−7.64	2.22e-14	0.991
GTExv8.EUR.Skin_Sun_Exposed_Lower_leg	*CLPTM1L*	5	rs452384	−7.61	2.84e-14	0.993
GTExv8.EUR.Whole_Blood	*IRF4*	6	rs12203592	12.36	4.58e-35	1
SKIN.Melanocyte	*RBAK*	7	rs6463231	−6.63	3.25e-11	0.997
SKIN.Melanocyte	*MTSS1*	8	rs12541595	−7.10	1.23e-12	0.999
SKIN.Melanocyte	*MOB3B*	9	rs1888379	5.31	1.10e-07	0.962

Results are displayed for SNP-gene pairs where the TWAS *P* is < 1.2e-06 and the colocalisation (COLOC) prior probability for model 4, colocalised (PP4), is ≥ 0.75 (see “Methods”). TWAS Z-score indicates the UM risk (positive Z-score) or protection (negative Z-score) inferred from over-expression of the candidate gene. The skin and whole blood tissues are from GTEx v8 European samples [40]. Skin melanocyte samples are from Zhang et al. [41].

## Results

**Fig. 1:**
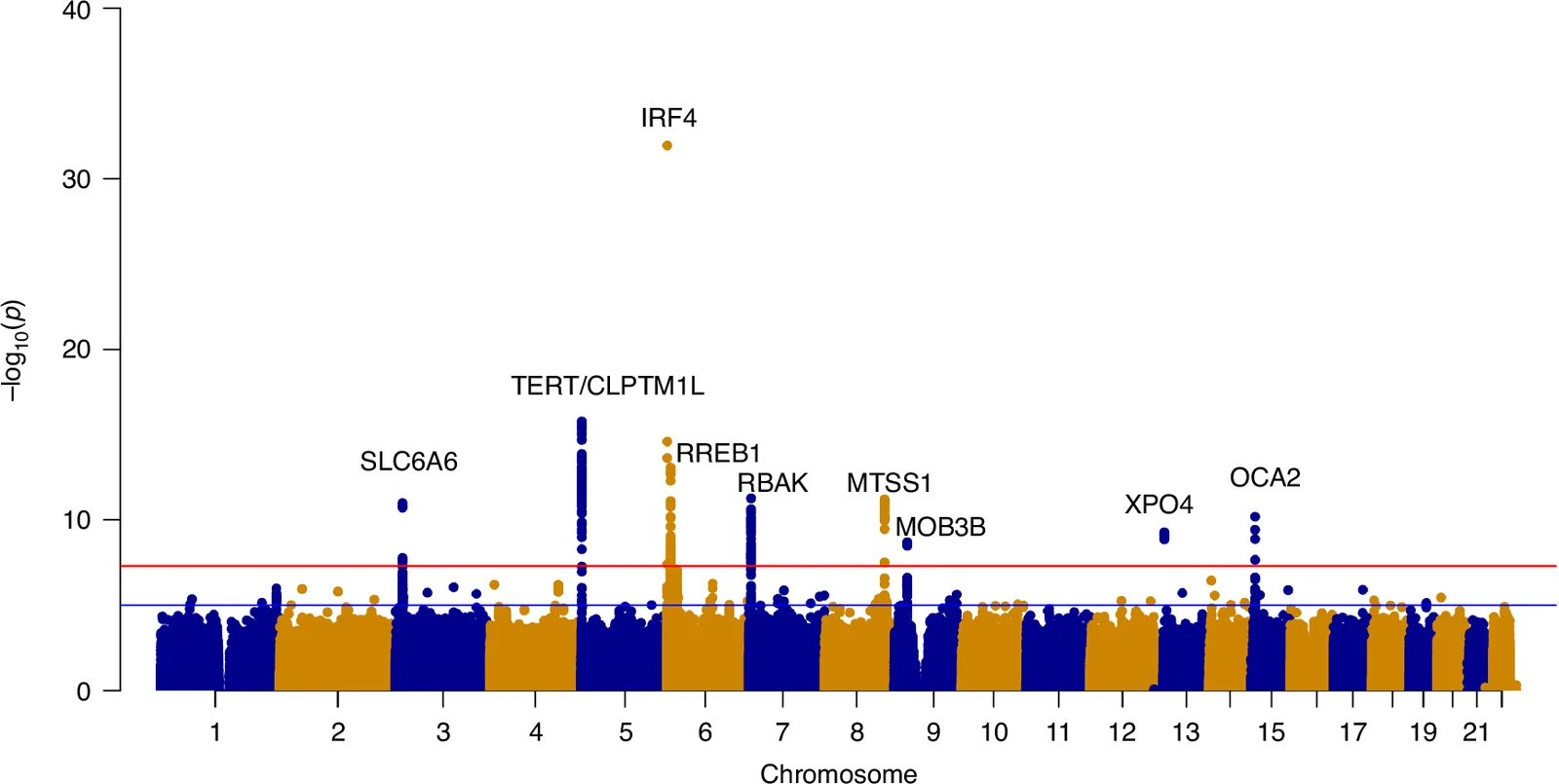
Manhattan plot for the fixed-effects inverse variance weighted meta-analysis of GWAS. The *X*-axis is the position along chromosomes, and the *Y*-axis is the negative log_10_ transformation of meta-analysis *P* values after correction based on the LDSC intercept. Each point represents a single SNP. A quantile-quantile (Q-Q) plot of corrected GWAS *P* values is also available in the supplement (Fig. s2).

**Fig. 2:**
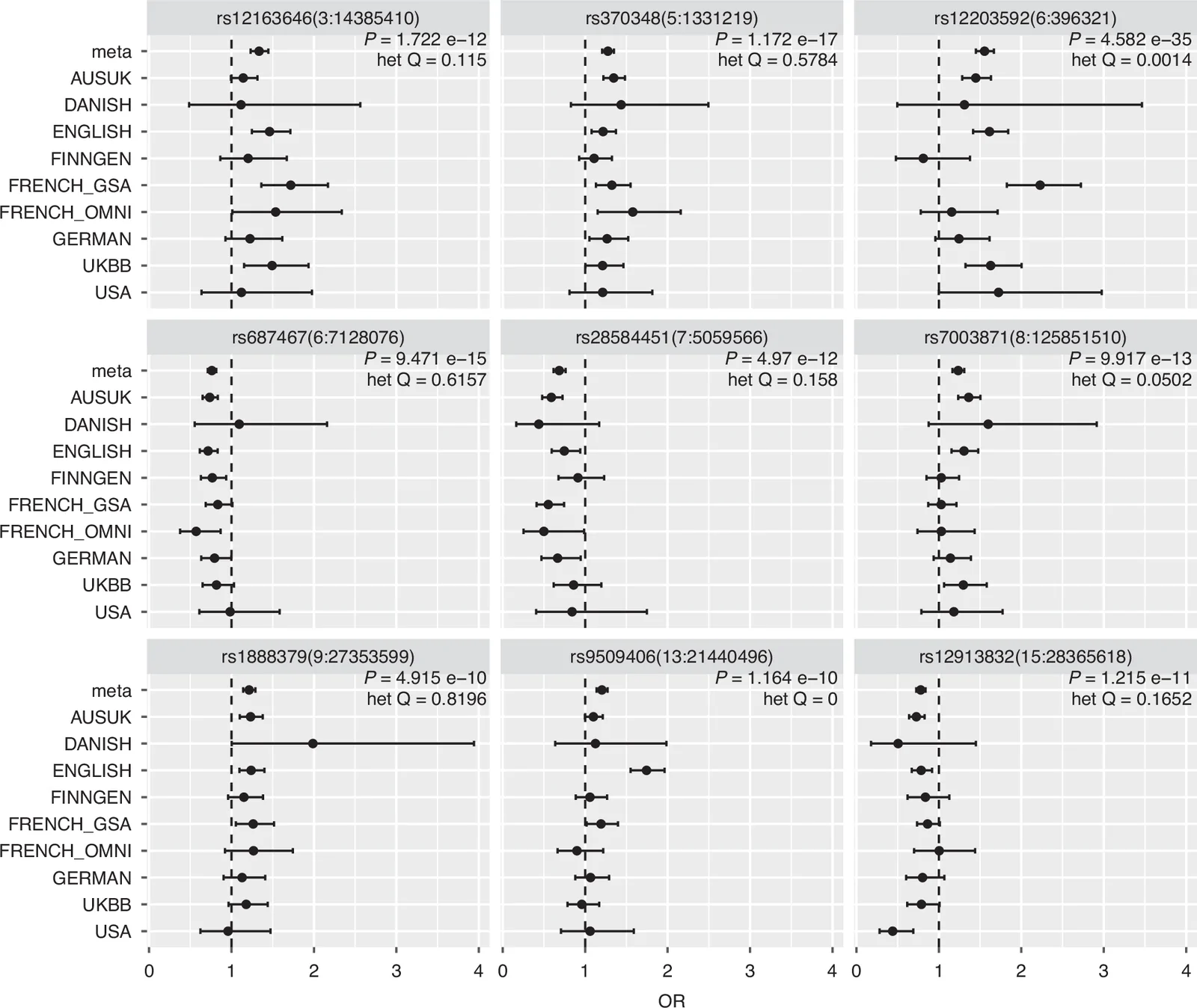
Forest plots for lead SNPs from loci associated with uveal melanoma. Odds ratios (OR) are plotted with the whiskers representing the 95% confidence interval. Study cohorts are named as in Table 1. Meta is the fixed effects meta-analysis estimate.

By combining 5839 UM cases with almost 350,000 controls (Methods; Table 1), we identify nine linkage disequilibrium (LD)-independent risk loci with SNPs achieving a fixed-effects genome-wide significance threshold of 5e-08 (Table 3, Figs. 1 and 2 and Supplementary Fig. s2). Three are novel to previous UM GWAS [28–31]. There is some evidence of residual inflation (LD score intercept 1.077; ratio with genomic inflation 0.74; Supplementary Table s1), so we have adjusted the test statistics and *P* values based on the LDSC intercept (Table 3). Conditional and joint analysis in GCTA-COJO does not identify any secondary signals at these loci. To elucidate the likely functional genes at each locus, we employed TWAS analysis using five expression quantitative trait loci (eQTL) datasets from the Genotype-Tissue Expression (GTEx) project [40], and one eQTL dataset from human melanocytes [41]. Based on a TWAS *P* value of 1.2e-06 and a COLOC PP4 > 0.75, we identify potential target genes for five out of these nine loci (Table 2). SNP-based heritability on the liability scale is 2.4% (SE 0.009), indicating our meta-analysis captures a significant (albeit modest) proportion of UM’s heritability.

**Table 3:** Lead SNPs for independent risk loci identified for UM

CHR	BP	SNP	Gene	EA	NEA	EA_freq	*P*	P(R)	SE	SE (*R*)	OR (95% CI)	OR(R) (95% CI)	*Q*	*I*^2^	Corrected *P*
3	14,385,410	rs12163646	*SLC6A6*	A	G	0.109	1.72 e-12	2.42e-07	0.041	0.058	1.33 (1.23 to 1.45)	1.35 (1.21 to 1.52)	0.115	38.0	1.06e-11
5	1,331,219	rs370348	*TERT/CLPTM1L*	G	A	0.448	1.17e-17	1.17e-17	0.028	0.028	1.28 (1.21 to 1.35)	1.28 (1.21 to 1.35)	0.578	0	1.66e-16
6	396,321	rs12203592	*IRF4*	T	C	0.131	4.58e-35	1.05e-07	0.036	0.077	1.55 (1.45 to 1.67)	1.50 (1.29 to 1.75)	0.001	68.4	1.11e-32
6	7,128,076	rs687467	*RREB1*	A	G	0.268	9.47e-15	9.47e-15	0.035	0.035	0.76 (0.71 to 0.82)	0.76 (0.71 to 0.82)	0.616	0.00	8.39e-14
7	5,059,566	rs28584451	*RBAK*	T	C	0.103	4.97e-12	2.31e-07	0.055	0.072	0.69 (0.62 to 0.76)	0.69 (0.60 to 0.79)	0.158	32.5	2.83e-11
8	125,851,510	rs7003871	*MTSS1*	C	T	0.329	9.92e-13	9.46e-05	0.029	0.046	1.23 (1.16 to 1.31)	1.20 (1.09 to 1.31)	0.050	48.4	6.33e-12
9	27,353,599	rs1888379	*MOB3B*	G	T	0.43	4.92e-10	4.92e-10	0.031	0.031	1.21 (1.14 to 1.29)	1.21 (1.14 to 1.29)	0.820	0.00	2.03e-09
13	21,440,496	rs9509406	*XPO4*	A	G	0.471	1.16e-10	0.1407	0.029	0.083	1.20 (1.14 to 1.27)	1.13 (0.96 to 1.33)	0	85.1	5.32e-10
15	28,365,618	rs12913832	*OCA2*	A	G	0.335	1.22e-11	5.15e-07	0.037	0.049	0.78 (0.73 to 0.84)	0.78 (0.71 to 0.86)	0.165	31.6	6.51e-11

CHR and BP are chromosome and base-pair position, hg19 build. SNP is the rsID for the lead SNP for a given locus. EA is the effect allele, NEA is the non-effect allele. Fixed effects (*P*) and random effects [P(R)] meta-analysis *P* values are reported. Odds ratio (OR), 95% confidence interval (95% CI) and standard error (SE) of the log(OR) are reported for the fixed effects and random effects (*R*) models. Finally, heterogeneity measures *Q* and *I*^2^ are provided. EA_freq is the effect allele frequency estimated from an HRC reference dataset. Corrected P is the *P* value after correction to the fixed-effects standard error based on the LDSC intercept of 1.077. Regional association plots for each locus are represented in Supplementary Fig. s3a–i).

### Previously reported UM risk loci

We identify nine UM risk loci (Table 3 and Supplementary Fig. s3a–i), confirming six previously reported loci. These include three loci first reported by Mobuchon et al. [30] and replicated by Mies et al. [31] (*TERT/CLPTM1L*, Fig. s3b; *IRF4*, Fig. s3c; and *OCA2*, Fig. s3i), and three loci first reported by Mies et al. *(RP11-53616.1*—our *SLC6A6* signal—Fig. s3a; *RREB1*, Fig. s3d; and *XPO4*, Fig. s3h). Most notably, the pigmentation genes *IRF4* and *OCA2* are replicated, further supporting the link between blue eye colour and UM risk (rs12913832 is the main determinant of blue/brown eye colour in Europeans) [27, 43, 44]. Effect sizes for these six loci are comparable to previous GWAS, and effect directions are consistent (Table s2). *TDP1*, reported as the top hit but not genome-wide significant in Thomsen et al. [30], and *IP6K1* (reported in Mies et al.) both fail to reach genome-wide significance in our meta-analysis. A more detailed comparison between studies is in the supplementary results section.

TWAS does not nominate target genes at any previously reported loci apart from *IRF4* and *TERT/CLPTM1L* (Table 2). For the remaining loci, we investigate publicly available eQTL datasets and nearby genes. rs687467 on chromosome 6 is within an intron of *RREB1*, which is implicated in multiple cancer types [45]. This SNP is also an eQTL for *SSR1* in blood [46]. rs9509406 on chromosome 13 is an eQTL for a range of genes across multiple tissues in GTEx, including *IL17D*, *IFT88*, *XPO4*, and *SLC35E1P1*, making nominating a specific candidate gene difficult [46]. Finally, in the absence of other functional data, the nearest gene to rs12163646 on chromosome 3 is *SLC6A6*. Notably, many of these genes have established links to oncogenic processes and/or metastasis suppression.

### Novel risk loci for UM

TWAS analysis supports *RBAK* (chromosome 7, lead SNP rs28584451, Fig. s3e), *MTSS1* (chromosome 8, lead SNP rs7003871, Fig. s3f), and *MOB3B* (chromosome 9, lead SNP rs1888379, Fig. s3g) as functional targets for three novel risk loci (Methods; Table 2). *RBAK* is involved in several crucial oncological functions, including DNA repair, cell-cell adhesion, and regulation of the Wnt and Hippo signalling pathways [47]. *MTSS1* encodes a metastasis suppressor protein, the reduced expression of which is observed in multiple cancer types [48]. *MOB3B* is part of the monopolar spindle-one-binder protein family, a group of genes that seemingly influence certain cancers via interactions with NDR/LATS kinase genes and subsequent regulation of the Hippo signalling pathway [49].

### Genetic correlation with melanoma-related traits

Genetic correlation analyses investigate the degree of genome-wide similarity and inferred risk between separate traits. There is an established phenotypic association between UM and CM, skin colour, eye colour, and naevus count, and there is evidence that the association is genetic; CM and UM share the same lead risk SNPs at *TERT/CLPTM1L*, *IRF4*, and *OCA2*. We therefore selected these traits for genetic correlation analyses. UM is significantly genetically correlated with CM (rg = 0.31, *P* = 0.01, Supplementary Table s3). UM shows no genetic overlap with skin colour (rg = −0.06, *P* = 0.37), and eye colour GWAS that are available to us do not attain sufficient statistical power. While not formally significant (*P* = 0.08), the point estimate of correlation between UM and cutaneous naevus count is similar to that observed with CM (rg = 0.25; CIs span the CM correlation estimate). Collectively, these results suggest that the genetic overlap between CM and UM may lie more in processes involved in melanocytic growth/senescence than other risk factors for melanoma, such as susceptibility to UV damage.

## Discussion

UM is the second-most common type of melanoma after CM, accounting for 4–5% of all melanomas. Somatic aberrations in UM are distinct from those observed in CM, relying more on activation of the G-protein-coupled signalling pathway rather than the MAPK pathway. However, regarding genetic predisposition, there appears to be more in common between these two histological subtypes, with high-penetrance germline variants in *CDKN2A*, *BAP1* and *POT1* contributing to both UM and CM susceptibility [20, 50, 51]. To further elucidate loci underlying UM predisposition, we carried out the largest GWAS meta-analysis of UM to date. Our meta-analysis includes more than twice the number of UM cases as the most recent GWAS conducted by Mies et al [31], which included four cohorts: the Wills Eye Hospital cohort (401 new cases and 762 controls), Thomsen et al. [29], Mobuchon et al. [30], and FinnGen [35]. We included the latter three cohorts (referred to in our study as “English”, “French (GSA)”, “French (Omni5)”, and “FinnGen”, respectively), but not the Wills Eye Hospital cohort. In addition to replicating previously established risk loci in *TERT/CLPTM1L*, *IRF4*, *OCA2*, *RP11-53616.1* (*SLC6A6*), *RREB1* and *XPO4*, we describe three novel risk loci and identify potential target genes involved in tumour suppression, metastasis, or cell cycle control.

TWAS suggests that *RBAK* expression in skin melanocytes is associated with rs28584451 (Table 2). *RBAK* encodes the retinoblastoma (RB)-associated *KRAB* zinc finger protein, a transcriptional repressor that partners with the RB tumour suppressor and acts as a key regulator of cell cycle control [52, 53]. TWAS also supports *MTSS1* as the candidate gene for the chromosome 8/rs7003871 locus. This gene is involved in regulating the actin cytoskeleton and is involved in the development and metastasis of multiple cancer types, including CM [54–56]. For lead SNP rs1888379 on chromosome 9, the candidate gene as defined by TWAS is *MOB3B*—a gene implicated in tumour suppression via regulation of the Hippo signalling pathway, and a noted risk locus for ocular melanosis (OM) in dogs [57, 58]. OM is one of the strongest phenotypic risk factors for UM development and metastasis in humans [59].

For those loci without TWAS support, publicly available eQTL data and genes proximal to the lead SNPs reveal several candidates with potential roles in UM development. For chromosome 3 lead SNP rs12163646, the nearest gene is *SLC6A6*. This gene encodes a solute carrier responsible for taurine transport across the cell membrane, and it has been linked to poor prognosis in a number of cancer types [60]. rs9509406 on chromosome 13 is an eQTL for multiple genes, including *IFT88*, *XPO4*, *IL17D*, and *EEF1AKMT1*, in a range of tissues. Of particular interest is *XPO4*, a tumour-suppressor gene responsible for nuclear export of EIF5A2 and SMAD3 [61]. EIF5A2 upregulation has been associated with cancer development and metastasis, while SMAD3 has shown a complex interplay with the MAPK pathway wherein MAPK mediates SMAD3 activity, which subsequently mediates TGF-beta1 signalling, affecting cell proliferation and differentiation [62–64].

For other loci, the link to UM development is less clear. rs687467 highlights an intriguing candidate gene in the zinc finger transcription factor *RREB1*, which influences a number of different oncogenic processes, including DNA damage repair, transcriptional regulation, and cell growth, proliferation and differentiation. This has led to observed direct links to multiple cancer types and provides clues to its possible role in UM [45].

Two loci, rs12202592 on chromosome 6 (*IRF4*) and rs9509406 on chromosome 13 (*XPO4*), show significant heterogeneity of effect sizes across the individual GWAS (Table 3). While the T allele of rs12202592 is associated with UM risk across cohorts (except FinnGen, where the allele is protective with wide confidence intervals), there is significant variation in the specific odds ratio (Fig. 2). This same allele has been shown to have an age-specific effect on naevus count [65]. While we lack the individual-level data to formally test for age-stratified effects in the meta-analysis, it is possible that a similar process is impacting its association with UM. The rs9509406 locus on chromosome 13 is primarily driven by a single UK-derived dataset (Fig. 2). Larger meta-analyses would be required to more accurately determine this SNP’s effect size for UM.

Our work has a number of strengths. This meta-analysis represents the largest study of genetic risk factors for UM to date. Internationally observed standards for diagnosis and treatment of UM are implemented in ophthalmic oncology clinics worldwide, and with the engagement of leading global ocular oncology clinics in addition to cancer registry data, we are assured of the accuracy of UM diagnoses in our study. We have respected this with stringent genotyping and analytical quality control. Through TWAS, we report multiple potential biological targets with previously demonstrated roles in tumorigenesis and/or metastatic suppression. We also assess heritability and provide UM genetic correlation estimates for CM, skin colour and naevus count based on the largest sample yet for UM genetic research.

We also acknowledge limitations in our study. There is notably some evidence of inflation within our data, presumably from residual stratification either between or within individual datasets, so we applied LDSC intercept-based correction to the test statistics. The heterogeneity in the *IRF4* and *XPO4* effect size estimates also warrants further examination, but they do appear to be genuine risk loci. The direction of effect for *IRF4* is consistent across studies, but the association for *XPO4* is largely driven by a single dataset and requires larger GWAS to better determine its true effect size for UM. As is the case with all GWAS, some loci do not have TWAS or eQTL support for candidate genes, and our highlighted nearby genes of interest should be treated more cautiously. Finally, due to its rarity in other populations, our meta-analysis only includes European ancestry samples, so it is unclear how the results would translate to non-European populations.

In conclusion, through the analysis of 5839 UM cases and 349,863 controls, we report nine risk loci for UM and a SNP-based heritability estimate of 2.4%. These loci highlight genes involved in tumour suppression, metastasis, or cell cycle control. Further in vitro studies of these novel loci are required to further characterise the mechanism of their influence on UM risk. We also report a significant genetic correlation of rg = 0.31 with CM using a prior CM GWAS including 30,134 cases and 81,415 controls [66]. While not formally significant, the point estimate of genetic correlation between UM and naevus count is similar to that seen for CM, suggesting that the common risk pathway for these two cancers is abnormal clonal expansion of melanocytes. Additionally, while only suggestive, this correlation is (from a biological perspective) consistent with a presumed 10–20% of UM arising from a pre-existing naevus [67], and approximately 30% of CM likewise arising from a pre-existing cutaneous naevus [68].

## Supplementary information


Online-Only Supplements
Figure s1: Regional association plots for TWAS analysis of uveal melanoma loci
Figure s2: Quantile-quantile (Q-Q) plot of GWAS P values after genomic control correction
Table s1: UM GWAS LD score intercept heritability estimates.
Figure s3a-i: Region plots for uveal melanoma risk loci
Table s2: Confirmation of seven UM risk loci reported by Mies et al.
Table s3: Genetic correlation estimates between uveal melanoma and associated phenotypic traits


## Data Availability

This study did not generate any novel or custom code – all analyses were performed with the published software packages as outlined in the Methods and Supplementary Methods, using specific filters and settings as described. Summary statistics of the uveal melanoma meta-analysis are available online (https://doi.org/10.5281/zenodo.20046892). The melanoma GWAS data used for the genetic correlation analysis are available on the database of Genotypes and Phenotypes (dbGAP, https://www.ncbi.nlm.nih.gov/gap), accession number phs001868.v1.p1. UK Biobank data (used to perform the skin colour GWAS) is available via application at https://www.ukbiobank.ac.uk/enable-your-research/register. The naevus count GWAS is available at https://doi.org/10.5281/zenodo.18537530. eQTL data from human melanocytes are available via dbGAP, accession number phs001500.v1.p1. GTEx v8 data is available via the GTEx data portal (https://www.gtexportal.org/home/downloads/adult-gtex/qtl). The CARTaGENE data is available for access requests on the study website https://cartagene.qc.ca/.
