# Supplementary material for: GWAS meta-analysis provides new insights into uveal melanoma risk
Source: Br J Cancer. 2026 Jun 16;135(6):983–91. doi: 10.1038/s41416-026-03499-7 (PMC13534482; doi:10.1038/s41416-026-03499-7)
Supplement: Supplementary file 3 [file 41416_2026_3499_MOESM5_ESM.pdf]

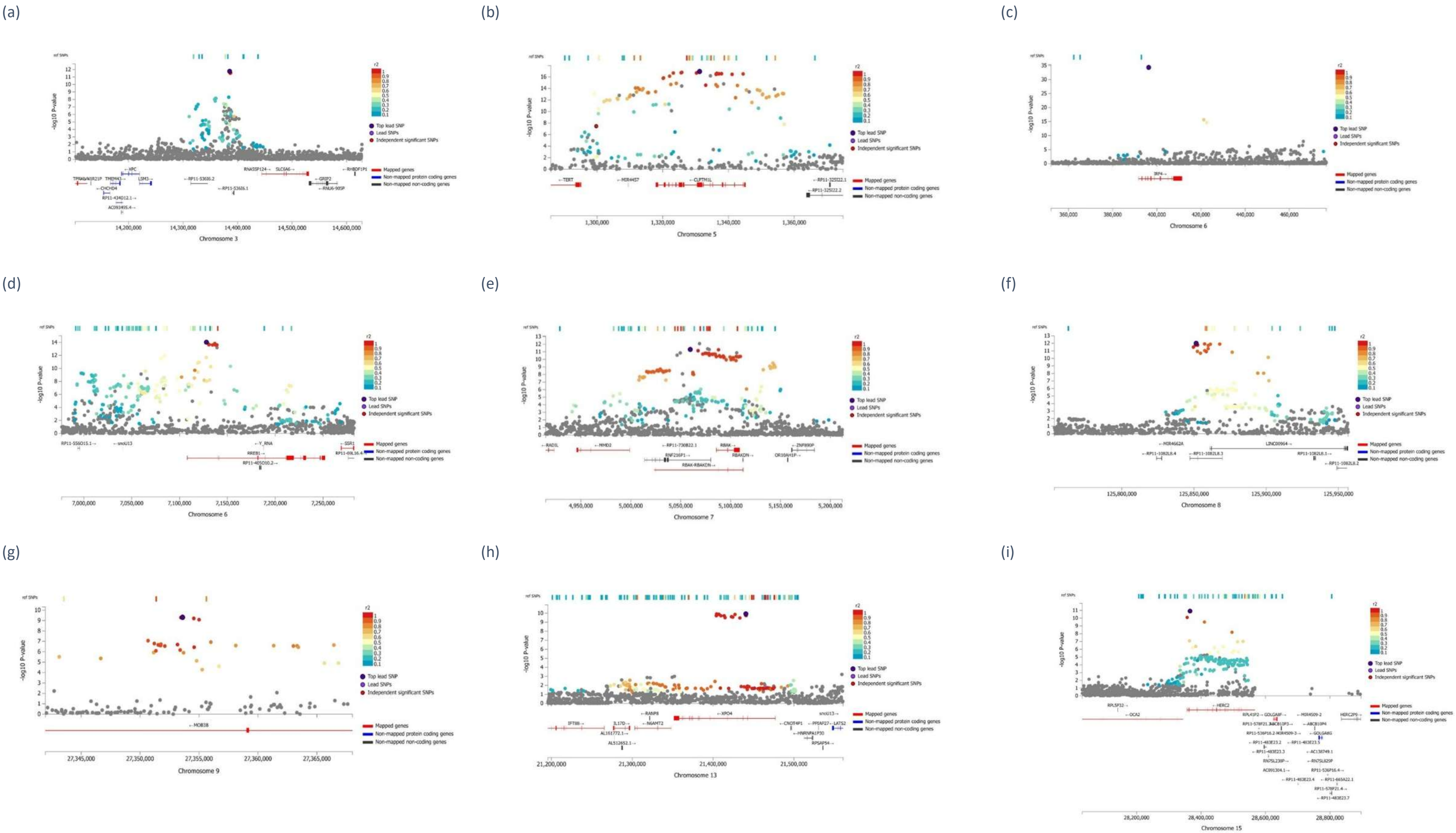

Figure s3a-i: Region plots for uveal melanoma risk loci
