# Supplementary material for: GWAS meta-analysis provides new insights into uveal melanoma risk
Source: Br J Cancer. 2026 Jun 16;135(6):983–91. doi: 10.1038/s41416-026-03499-7 (PMC13534482; doi:10.1038/s41416-026-03499-7)
Supplement: Supplementary file 4 [file 41416_2026_3499_MOESM1_ESM.docx]

**Online-Only Supplements**

**Methods**

LDSC regression and heritability estimate

Using European LD scores from the 1000 Genomes Project and a reference SNP list from HapMap 3, SNP heritability (h²_snp_) and genomic inflation factors were determined via LD score (LDSC) regression [1]. This was performed for each GWAS dataset as well as the final meta-analysis. For conversion of h²_snp_ from the observed scale to the liability scale, a population prevalence of 5.3e-04 was calculated using UM incidence in the UKBB. To assess the impact of observed genomic inflation on *P* values, SNP effect estimate standard errors were multiplied by the LDSC intercept and *P* values recalculated.

Functional mapping and annotation

Locus fine-mapping and functional SNP annotation were performed using the online platform FUMA GWAS (<https://fuma.ctglab.nl/>) [2], using 1000 Genomes European samples as the reference population. Lead SNPs were distinguished from independent SNPs based on an LD R² threshold of 0.05, and loci were merged into LD blocks of a maximum of 250kb.

Genetic correlation

LDSC was used to determine the global genetic correlation, which is a parameter that describes the genetic relationship between two traits and can reflect the shared biological pathways and/or the causal relationships between traits [3]. Using publicly available summary-level statistics, this study investigated the genetic correlation between UM and CM (using a clinically confirmed GWAS of 30,134 cases and 81,415 controls) [4], cutaneous naevus count (using a GWAS of 85,967 individuals) [5], and skin colour from the UK Biobank (N = 433,288) [6].

Queensland cohort

Patients participating via the Queensland Ocular Oncology Service, Terrace Eye Centre, Brisbane, were recruited to the study following a diagnosis of primary UM and with informed consent. All participants were examined, and UM diagnoses were confirmed by an expert Ocular Oncologist (LAM, SKW, TB, WJG). UM was confirmed histologically if tissue was available. Germline DNA was extracted from saliva or peripheral blood via the salting out method, purified using prepIT.L2P (DNA Genotek, Inc.), and quantified using the NanoDrop Microvolume UV-Vis Spectrophotometer (Thermo Fisher Scientific). Genotyping of Queensland DNA samples was performed at the Australian Translational Genomics Centre (ATGC) and the Australian Genome Research Facility (AGRF).

Western Australia cohort

Patients were recruited from Royal Perth, Sir Charles Gairdner, or Fiona Stanley Hospitals in Western Australia under approved Human Research Ethics Committee protocols from Edith Cowan University (No. 11543 and No. 18957) and Sir Charles Gairdner Hospital (No. 2013-246 and No. RGS0000003289). Patients were diagnosed clinically by an expert ophthalmologist (TWI). Germline DNA was extracted from buffy coat or peripheral blood mononuclear cells using the MagMax DNA Multi-Sample Ultra 2.0 Kit (Thermo Fisher Scientific) on the KingFisher Duo Prime (Thermo Fisher Scientific) or QIAamp DNA Mini Kit (Qiagen), respectively. DNA was quantified using an NP80 UV-Vis Spectrophotometer (Implen).

English cohorts

All UM patients from the Liverpool cohort were diagnosed in the ocular oncology centre of the Royal Liverpool University Hospitals Group, UK. All patients had provided consent to take part in research as part of the University of Liverpool ethically approved Ocular Oncology Biobank (Research Ethics Committee Ref 21/NW/0139). A Material Transfer Agreement was put in place between the University of Liverpool and QIMR Berghofer Medical Research Institute for this study. DNA was extracted from EDTA whole blood on the Qiagen QIAsymphony using the DSP DNA Midi extraction kit. Previously published summary statistics are included (English, Table 1) [7]. Additional newly collected samples were genotyped and analysed in combination with additional Australian and UK samples (AUSUK, Table 1).

Scottish cohort

Patients of the Scottish Ocular Oncology Service located at Gartnavel General Hospital, Glasgow, were consented and recruited to the study following a diagnosis of primary uveal melanoma (Research Ethics Committee Ref 19/WA/0306). Germline DNA was extracted from saliva and then purified using prepIT.L2P (DNA Genotek, Inc.) at the University of the Highlands and Islands. DNA samples were transferred to the QIMR Berghofer, and genotyping was performed at the AGRF.

German (Essen) cohort

All UM patients from the Essen cohort were diagnosed and recruited in the ophthalmology department of the University Hospital Essen, Germany. DNA was extracted from EDTA blood using Nucleon BACC Genomic DNA extraction Kit (Amersham), EZ1 DSP DNA Blood Kit and FlexGene DNA Whole Blood Kit (Qiagen). Ethical approval was obtained by the ethics committee from the Medical faculty of the University Duisburg-Essen (project approval number 19-8605-BO).

American cohort

All UM patients from the Ohio State University cohort were diagnosed or referred to the Department of Ophthalmology and Visual Sciences at the Ohio State University Wexner Medical Center. DNA was extracted from either blood buffy-coat using the Puregene DNA extraction kit (Qiagen) or from saliva samples using the Norgen Saliva DNA collection and isolation kits (Norgen Biotek, Thorold, Ontario, Canada). Institutional Review Board Approval was obtained from The Ohio State University Office of Research Responsibility and Compliance, protocol number 2006C0045.

Danish cohort

All UM patients from the Danish cohort were diagnosed at the Department of Ophthalmology, Rigshospitalet, Copenhagen, Denmark, and have given informed consent. Ethics approval was obtained from the National Ethics Committee, protocol number 1905114. DNA extraction was performed by the Capitol regional Bio- and genome-bank from stored whole blood samples.

French cohorts

The study was approved by the Ethical Committee and Internal Review Board at the Institut Curie, Paris, including consecutive patients diagnosed with UM and who consented to participate in the study. Three series of genomic DNA extracted for blood of UM patients were genotyped at the *Centre National de Génotypage* (Evry, France), the first series (Dataset 1) of 279 cases using Illumina HumanOmni5 platform, the second and third series (Datasets 2 and 3) of 369 and 577 cases, respectively, on the Infinium Global Screening Array 24 v1.0 platform. Genotypes were called using default parameters in GenomeStudio (Illumina). GWAS using these UM series were previously reported [8,9].

QSkin

The QSkin Sun and Health Study recruited over 43,000 people with informed consent from the general population of Queensland, Australia, using electoral roll data in 2011. 19,119 of these participants provided a saliva sample for DNA extraction, and their DNA was genotyped using the GSA [Illumina, San Diego, USA]. A follow-up phase of recruitment in 2019 collected (again with informed consent) an additional 8,296 people Australia-wide who voluntarily provided a saliva sample for DNA extraction; this second phase of volunteer samples were then handled as per the earlier phase. Comprehensive details of participant recruitment methods have been previously published [10]. Samples from QSkin were used as healthy controls and were cleaned and imputed as described in the main methods section. Ethics approval was managed by the Human Research Ethics Committee of QIMR Berghofer (P1309, P2304, and P3434).

UK Biobank

For UKBB, samples were filtered to include only participants of European ancestry using the first two principal components compared to a European reference set of self-reported ‘white-british’ samples within UKBB. Samples were also removed if they had genotype vs reported sex mismatch, if they had a SNP missingness greater than 3%, or if they showed extreme heterozygosity. SNPs were excluded if they deviated from Hardy-Weinberg Equilibrium (*P* value < 1e-12), had a high call rate < 98%, a minor allele frequency < 0.01, or were poorly imputed (INFO < 0.3). UM case status for UKBB participants was determined using cancer registry data ICD9 code - 1906, ICD10 code - C69.3 (malignant neoplasm of the choroid; n = 188) or ICD10 code - C69.4 (malignant neoplasm of the ciliary body; n = 23), to give a total post-QC sample size of 211 UM cases. Controls were cancer-free, including being free from keratinocyte cancers. Sex, age and the first 10 principal components were included as covariates during GWAS analysis.

FinnGen

QC and GWAS analysis protocols for FinnGen have been previously published [11]. Cases were identified from a hospital or death record for ICD10 codes C69.3 or C69.4. Summary statistics for 242 cases and 299,884 cancer-free controls were accessed from Release 9. Full details of the GWAS methods are accessible here <https://finngen.gitbook.io/documentation/methods/phewas>

Software versions and links

PLINK v1.90b6.8 (<http://pngu.mgh.harvard.edu/purcell/plink/>)

PLINK v2.00a5LM (<http://pngu.mgh.harvard.edu/purcell/plink/>)

GCTA v1.94.1 Linux (<https://yanglab.westlake.edu.cn/software/gcta/>)

R version 3.6.2 ([https://www.R-project.org/](https://www.r-project.org/))

TWAS/Fusion (<http://gusevlab.org/projects/fusion/>)

FUMA (<https://fuma.ctglab.nl/>) accessed 15 Apr 2025

LD Score Regression (LDSC) Version 1.0.1 (<https://github.com/bulik/ldsc>)

**Results**

We confirm six known UM risk loci: *RP11-53616.1* (*SLC6A6* signal, chromosome 3, lead SNP rs12163646, Figure s3a), *TERT*/*CLPTM1L* (chromosome 5, lead SNP rs370348, Figure s3b), *IRF4* (chromosome 6, lead SNP rs12203592, Figure s3c), *RREB1* (chromosome 6, lead SNP rs687467 (eFigure s3d), *XPO4* (chromosome 13, lead SNP rs9509406, Figure s3h), and *OCA2* (chromosome 15, lead SNP rs12913832, Figure s3i). While rs370348 is proximal to the *TERT* gene, our TWAS analysis suggests that the functional gene may be *CLPTM1L* (Table 2 and Figure s1). *CLPTM1L* was established as a UM risk locus by Mobuchon et al. in 2017, with rs421284 as the lead SNP (OR = 1.7, CI 1.43 to 2.05, *P* = 5e-09) [8]. Our lead SNP for *CLPTM1L* (rs370348) shows consistent effect directionality (OR = 1.3) and a stronger association (*P* = 1.17e-17), and is also in perfect LD with rs421284. *TDP1* was flagged as a potential risk locus for UM by Thomsen et al. (2020) with their lead SNP rs3759710 showing a suggestive *P* value of 1.07e-07 [7]. This locus is not replicated in our meta-analysis (*P* = 0.003) and the lowest *P* value across SNPs within 2Mb of rs3759710 is 5.52e-05. The *IP6K1* (rs9849038) locus from the 2025 GWAS by Mies et al also does not reach genome-wide significance and shows heterogeneity across studies in our meta-analysis (*P* = 4.59e-05, OR = 1.17, CI = 1.08 to 1.25, I^2^ = 81.02).

**References**

1) Bulik-Sullivan BK, Loh P-R, Finucane HK, Ripke S, Yang J, Schizophrenia Working Group of the Psychiatric Genomics Consortium, et al. LD Score regression distinguishes confounding from polygenicity in genome-wide association studies. Nat Genet. 2015 Mar;47(3):291–5.
2) Watanabe K, Taskesen E, van Bochoven A, Posthuma D. Functional mapping and annotation of genetic associations with FUMA. Nat Commun. 2017 Nov 28;8(1):1826.
3) Bulik-Sullivan B, Finucane HK, Anttila V, Gusev A, Day FR, Loh P-R, et al. An atlas of genetic correlations across human diseases and traits. Nat Genet. 2015 Nov;47(11):1236–41.
4) Landi MT, Bishop DT, MacGregor S, Machiela MJ, Stratigos AJ, Ghiorzo P, et al. Genome-wide association meta-analyses combining multiple risk phenotypes provide insights into the genetic architecture of cutaneous melanoma susceptibility. Nat Genet. 2020 May;52(5):494–504.
5) Jayasinghe GJMSR, Zhu G, Pandeya N, Olsen CM, Martin NG, Lind PA, et al. A large-scale genome-wide association meta-analysis for nevus count provides direct insights into the genetics of melanoma [Internet]. medRxiv. 2025. p. 2025.02.25.25322831. Available from: http://medrxiv.org/content/early/2025/02/25/2025.02.25.25322831.abstract
6) Seviiri M, Law MH, Ong J-S, Gharahkhani P, Fontanillas P, 23andMe Research Team, et al. A multi-phenotype analysis reveals 19 susceptibility loci for basal cell carcinoma and 15 for squamous cell carcinoma. Nat Commun. 2022 Dec 10;13(1):7650.
7) Thomsen H, Chattopadhyay S, Hoffmann P, Nöthen MM, Kalirai H, Coupland SE, et al. Genome-wide study on uveal melanoma patients finds association to DNA repair gene TDP1. Melanoma Res. 2020 Apr;30(2):166–72.
8) Mobuchon L, Battistella A, Bardel C, Scelo G, Renoud A, Houy A, et al. A GWAS in uveal melanoma identifies risk polymorphisms in the CLPTM1L locus. NPJ Genom Med [Internet]. 2017 Mar 10;2(1). Available from: http://dx.doi.org/10.1038/s41525-017-0008-5
9) Mobuchon L, Derrien A-C, Houy A, Verrier T, Pierron G, Cassoux N, et al. Different pigmentation risk loci for high-risk monosomy 3 and low-risk disomy 3 uveal melanomas. J Natl Cancer Inst. 2022 Feb 7;114(2):302–9.
10) Olsen CM, Green AC, Neale RE, Webb PM, Cicero RA, Jackman LM, et al. Cohort profile: the QSkin Sun and Health Study. Int J Epidemiol. 2012 Aug;41(4):929–929i.
11) Mbatchou J, Barnard L, Backman J, Marcketta A, Kosmicki JA, Ziyatdinov A, et al. Computationally efficient whole-genome regression for quantitative and binary traits. Nat Genet. 2021 July;53(7):1097–103.

**Figure and table legends**

**Figure s1: Regional association plots for TWAS analysis of uveal melanoma loci.** Panel contents and order as listed in Table 2. The negative log_10_ transformation of TWAS *P* values are plotted (Y-axis) against chromosomal position (X-axis) with the TWAS gene (or transcript) highlighted in green.

**Figure s2: Quantile-quantile (Q-Q) plot of GWAS P values after genomic control correction**

**Table s1: UM GWAS LD score intercept heritability estimates**. *Where the LD score intercept is greater than 1, the ratio of the genomic inflation to intercept is informative; 0.2 or lower is considered limited or negligible evidence of inflation.

**Figure s3a-i: Region plots for uveal melanoma risk loci.** From top left to bottom right: (a) chromosome 3, lead SNP rs12163646, (b) chromosome 5, lead SNP rs370348, (c) chromosome 6, lead SNP rs12203592, (d) chromosome 6, lead SNP rs687467, (e) chromosome 7, lead SNP rs28584451, (f) chromosome 8, lead SNP rs7003871, (g) chromosome 9, lead SNP rs1888379, (h) chromosome 13, lead SNP rs9509406, (i) chromosome 15, lead SNP rs12913832.

**Table s2:** **Confirmation of seven UM risk loci reported by Mies et al.** SNPs were first compared in a reduced meta-analysis involving non-overlapping cohorts only, and then in the full meta-analysis involving all cohorts. The reduced meta-analysis included five cohorts; AUSUK, German, USA, Danish and UKBB. The beta and *P* values from Mies et al. were extracted from the full summary data posted on GWAS catalog (study access: GCST90568461).

**Table s3**: **Genetic correlation estimates between uveal melanoma and associated phenotypic traits**
