# Supplementary material for: GWAS meta-analysis provides new insights into uveal melanoma risk
Source: Br J Cancer. 2026 Jun 16;135(6):983–91. doi: 10.1038/s41416-026-03499-7 (PMC13534482; doi:10.1038/s41416-026-03499-7)
Supplement: Supplementary file 5 [file 41416_2026_3499_MOESM4_ESM.docx]

| GWAS | Liability scale h^2^_snp_ | Lambda genomic inflation | LD score Intercept | Ratio* |
| --- | --- | --- | --- | --- |
| AUSUK | 0.029 (0.007) | 1.041 | 1.006 (0.006) | 0.131 (0.141) |
| German | -0.002 (0.025) | 1.017 | 1.015 (0.007) | 1.047 (0.455) |
| USA | -0.024 (0.054) | 1.023 | 1.022 (0.006) | 1.209 (0.331) |
| Danish | -0.148 (0.034) | 0.848 | 0.875 (0.005) | NA |
| French (GSA) | -0.007 (0.049) | 1.130 | 1.131 (0.007) | 1.010 (0.056) |
| French (Omni5) | 0.078 (0.043) | 0.975 | 0.955 (0.006) | NA |
| UKBB | 0.011 (0.012) | 1.005 | 0.984 (0.006) | NA |
| FinnGen | <0.001 (0.001) | 1.011 | 0.997 (0.007) | NA |
| English | 0.019 (0.026) | 1.026 | 1.021 (0.008) | 0.744 (0.274) |
| Meta-analysis | 0.024 (0.009) | 1.096 | 1.077 (0.007) | 0.740 (0.065) |

**Table s1: UM GWAS LD score intercept heritability estimates**
