# Supplementary material for: GWAS meta-analysis provides new insights into uveal melanoma risk
Source: Br J Cancer. 2026 Jun 16;135(6):983–91. doi: 10.1038/s41416-026-03499-7 (PMC13534482; doi:10.1038/s41416-026-03499-7)
Supplement: Supplementary file 6 [file 41416_2026_3499_MOESM6_ESM.docx]

| chr:bp | SNP | Locus | A1 | A2 | Mies et al. | | Reduced meta-analysis | | Full meta-analysis | | |
| --- | --- | --- | --- | --- | --- | --- | --- | --- | --- | --- | --- |
|  |  |  |  |  | beta | P | beta | P | beta | P | P_corrected |
| 3:14387519 | rs11707457 | RP11-53616.1/SLC6A6 | T | C | 0.3641 | 8.57E-11 | 0.1864 | 8.98E-04 | 0.2859 | 2.78E-12 | 1.65E-11 |
| 3:49771990 | rs9849038 | IP6K1 | A | G | 0.2982 | 6.57E-09 | 0.0272 | 5.93E-01 | 0.1532 | 4.59E-05 | 8.60E-05 |
| 5:1325590 | rs421284 | TERT/CLPTM1L | C | T | 0.2527 | 8.24E-11 | 0.2656 | 1.11E-11 | 0.2429 | 1.95E-17 | 2.67E-16 |
| 6:396321 | rs12203592 | IRF4 | T | C | 0.441 | 7.11E-19 | 0.3787 | 4.93E-15 | 0.4410 | 4.58E-35 | 1.11E-32 |
| 6:7136772 | rs551143 | RREB1 | A | G | -0.2964 | 1.84E-10 | -0.2535 | 2.02E-07 | -0.2700 | 1.48E-14 | 1.27E-13 |
| 13:21440496 | rs9509406 | XPO4 | A | G | 0.2578 | 1.90E-11 | 0.0675 | 9.11E-02 | 0.1847 | 1.16E-10 | 5.32E-10 |
| 15:28365618 | rs12913832 | HERC2/OCA2 | A | G | -0.3704 | 2.84E-15 | -0.3214 | 8.04E-10 | -0.2485 | 1.22E-11 | 6.51E-11 |

**Table s2:** **Confirmation of seven UM risk loci reported by Mies et al.**
