# Supplementary material for: GWAS meta-analysis provides new insights into uveal melanoma risk
Source: Br J Cancer. 2026 Jun 16;135(6):983–91. doi: 10.1038/s41416-026-03499-7 (PMC13534482; doi:10.1038/s41416-026-03499-7)
Supplement: Supplementary file 7 [file 41416_2026_3499_MOESM7_ESM.docx]

| Trait | Genetic Correlation (SE) with uveal melanoma | Z-score | P |
| --- | --- | --- | --- |
| CM | 0.312 (0.123) | 2.53 | 0.011 |
| Naevus count | 0.253 (0.145) | 1.75 | 0.081 |
| Skin pigmentation | -0.062 (0.069) | -0.90 | 0.367 |

**Table s3**: **Genetic correlation estimates between uveal melanoma and associated phenotypic traits**
